# Nature-Identical Compounds and Organic Acids Reduce *E. coli* K88 Growth and Virulence Gene Expression In Vitro

**DOI:** 10.3390/toxins12080468

**Published:** 2020-07-23

**Authors:** Andrea Bonetti, Benedetta Tugnoli, Barbara Rossi, Giulia Giovagnoni, Andrea Piva, Ester Grilli

**Affiliations:** 1 Dipartimento di Scienze Mediche Veterinarie (DIMEVET), Università di Bologna, via Tolara di Sopra 50, 40064 Ozzano dell’Emilia (BO), Italy; andrea.bonetti15@unibo.it (A.B.); giulia.giovagnoni4@unibo.it (G.G.); andrea.piva@unibo.it (A.P.); 2 Vetagro S.p.A., via Porro 2, 42124 Reggio Emilia, Italy; benedetta.tugnoli@vetagro.com (B.T.); barbara.rossi@vetagro.com (B.R.); 3 Vetagro Inc., 116 W. Jackson Blvd., Suite #320, Chicago, IL 60604, USA

**Keywords:** post-weaning diarrhoea, pigs, *Escherichia coli* K88, antibiotics, nature identical compounds, organic acids, virulence regulation, enterotoxins

## Abstract

Post-weaning diarrhoea (PWD) is one of the long-standing challenges in pig husbandry. Due to the risks of resistance caused by antibiotics (AB) misuse, conventional treatments against *Escherichia coli* K88 (*E. coli* K88), the PWD etiological agent, urgently need to be replaced. Organic acids (OA) and nature-identical compounds (NIC) are currently finding a central role in infection management thanks to their recognized antimicrobial activity. This study investigated the susceptibility of an *E. coli* K88 field strain to a wide panel of AB, NIC, and OA. Secondly, we evaluated the ability of sub-lethal doses of the most active compounds to modulate the expression of *E. coli* K88 virulence genes. Results showed that the bacterial strain was resistant to many of the tested antibiotics, but an antimicrobial action was registered for selected NIC and OA. The quantitative PCR analysis revealed that thymol, carvacrol, eugenol, and benzoic acid were able to downregulate (*p* < 0.05) the expression of bacterial genes related to motility, adhesion to enterocytes, heat-labile (LT) and heat-stable (ST) toxin secretion, quorum sensing, and biofilm formation. Therefore, this study demonstrated that selected OA and NIC not only control *E. coli* K88 growth but also modulate the expression of many virulence genes at sub-lethal doses, thus offering new insights on their mechanism of action and suggesting a powerful tool to manage PWD.

## 1. Introduction

Post-weaning diarrhoea (PWD) is one of the main economic losses in the pig breeding industry because of its large impact on the most pivotal phase of the pig production cycle. Even if several predisposing and contributing factors have been identified, the main etiological agent is represented by enterotoxigenic *Escherichia coli* (ETEC) [1]. These bacterial strains are considered to be the most significant global cause of severe watery diarrhoea in piglets, with side effects varying from growth retardation to sudden death [2]. 

The onset of PWD symptoms in pigs is primarily associated with *Escherichia coli* K88 (*E. coli* K88), the main representative amongst ETEC strains [3]. The bacterium can enter the digestive tract of weaning piglets via the oral route. From the upper jejunum to the ileum, *E. coli* K88 colonizes the intestinal mucosal surface employing its fimbrial adhesins, hair-like polymeric structures essential for ETEC pathogenicity and capable of binding to receptors on enterocytes [4].

However, the cornerstone of *E. coli* K88 pathogenicity lies in its ancillary plasmids, which harbour genes encoding for heat-labile (LT) and heat-stable (ST) toxins, which target intestinal cells. Although by different means, toxins activate signal transduction pathways that finally bring the severe intestinal secretion of electrolytes, disruptions of cellular tight-junctions, loss of water, and, consequently, diarrhoea [5,6].

Antibiotics (AB) represent one of the main approaches against PWD. However, scientists and political institutions are promoting action towards a reduction of their use, because of worries related to the continuous spread of antimicrobial resistance [7].

This issue, together with the consumer demand for novel molecules with antibacterial properties, have encouraged research towards alternatives, such as organic acids (OA) and nature identical compounds (NIC). These classes of compounds all have a recognized bactericidal effect at different concentrations against several pathogens [8,9].

Organic acids embody a wide group of organic compounds considered one of the most-used feed additives in pig nutrition [10]. OA have been proved to have antimicrobial effects because of their action on cellular metabolism and because of their ability to deplete cellular energy while triggering ATPase pumps to let H^+^ anions out of cells [11,12]. Interestingly, this bactericidal activity only occurs against pH-sensitive bacteria, such as bacteria belonging to *Escherichia, Clostridium, Salmonella*, and *Listeria* genus. Non-pH-sensitive bacteria, such as *Bifidobacteria* and *Lactobacilli*, can innately face and manage lower intracytoplasmic pH levels, without being negatively affected by OA anions [13].

Nature-identical compounds are pure chemicals which reflect molecules naturally occurring in essential oils, thus overcoming the intrinsic variability in their composition [14]. Their mode of action is related to their capacity to form pores on the bacterial membrane, induce cell lysis, and inhibit enzyme activity, in addition to anti-inflammatory and antioxidant activities [15,16,17].

This study aimed to evaluate the in vitro susceptibility of a field strain of *E. coli* K88 to selected antibiotics and antimicrobial compounds. More precisely, we investigated how AB, OA, and NIC could affect *E. coli* K88 growth, survival, and expression of various virulence genes involved in motility, quorum sensing, biofilm formation, adherence to cells, and LT and ST toxin production.

## 2. Results

### 2.1. Minimal Inhibitory Concentration (MIC) Assay

The results of the MIC assay are shown in Table 1. *Escherichia coli* K88 (*E. coli* K88) proved to be resistant to amoxicillin, ampicillin, lincomycin, neomycin, and penicillin G up to 64 mg/L. Doxycycline and colistin were effective at MIC values of 32 mg/L and 4 mg/L, respectively. Amongst nature-identical compounds, the most efficient were carvacrol and thymol, with MIC values registered at 1.87 mM, while eugenol proved to inhibit bacterial growth at a concentration of 3.75 mM. Many of the tested OAs failed to report an MIC value up to the highest tested concentration; only hexanoic acid showed the capacity to completely prevent bacterial proliferation at 25 mM, while benzoic acid and sorbic acid exerted the same action at 50 mM.

### 2.2. Minimal Bactericidal Concentration (MBC) Assay

**Figure 1:**
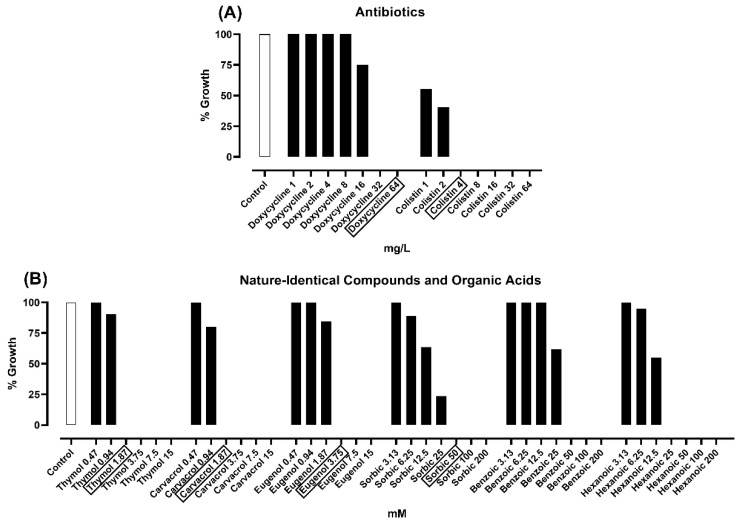
*Escherichia coli* K88 growth after 24 h incubation with antibiotics (**A**), nature-identical compounds or organic acids (**B**) that reported a minimal inhibitory concentration (MIC) value against the bacterial strain during the MIC assay. Growth is expressed as a percentage relative to the control (strain only); values are presented as means of three technical replicates. In rectangles are highlighted the minimal bactericidal concentration (MBC) of each substance; for benzoic and hexanoic acid, no MBC was found up to the highest tested concentration.

Figure 1 shows the results of the MBC assay. Doxycycline and colistin killed *E. coli* K88 at 64 mg/L and 4 mg/L, respectively. MBC values were the same as MIC values for thymol, carvacrol (both 1.87 mM), and eugenol (3.75 mM). Amongst OAs, only sorbic acid was able to kill the tested *E. coli* strain at 50 mM, whereas there was no MBC for benzoic acid and hexanoic acid up to the highest tested concentrations.

### 2.3. Gene Expression Analysis

**Figure 2:**
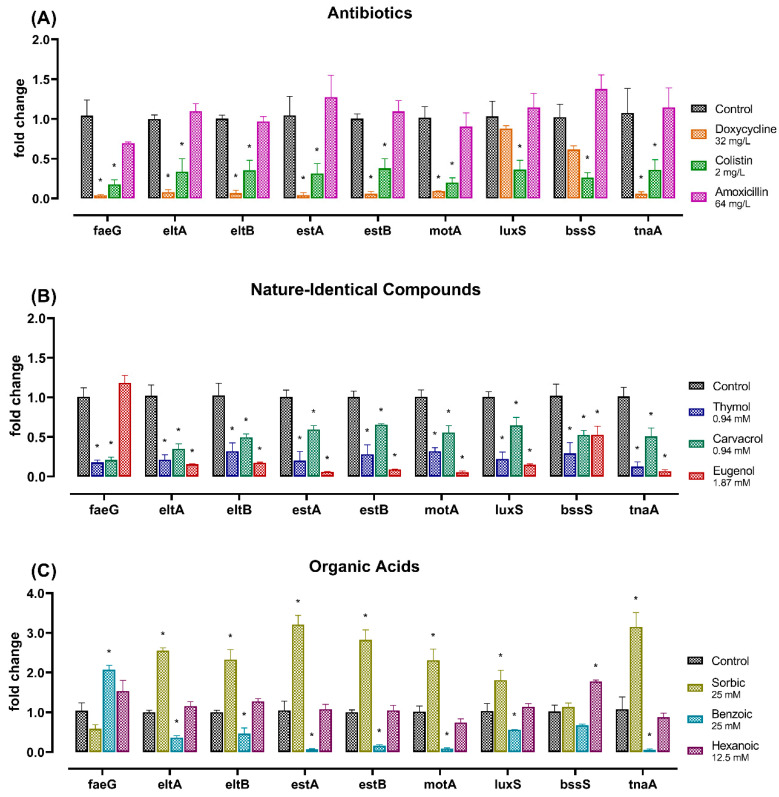
Effect of different antibiotics (**A**), nature-identical compounds (**B**), and organic acids (**C**) on relative expression levels of *E. coli* K88 virulence genes related to cellular adhesion (*faeG*), heat-labile toxin (*eltA* and *eltB*), heat-stable toxins (*estA* and *estB*), motility (*motA*), quorum sensing (*luxS*), and biofilm formation (*bssS* and *tnaA*). Data are expressed as means (n = 3) and SEM is represented by vertical bars. For each gene, significant differences between each substance and its control are marked by asterisks (*p* < 0.05).

The effects of all the selected AB, NIC, and OAs on the expression of virulence genes in the *E. coli* K88 strain employed in this study are presented in Figure 2A–C. Doxycycline and colistin reported a general downregulation for all the analyzed genes, except for *bssS*, that showed only a reduction trend (*p* < 0.1) in its expression level, and *luxS*, which was not significantly influenced by doxycycline. Amoxicillin did not show significant differences in mRNA levels (Figure 2A). Thymol, carvacrol, and eugenol significantly reduced the expression of all the genes tested, with the unique exception of *faeG* for eugenol, whose level did not differ (*p* > 0.05) from the untreated control (Figure 2B). Benzoic acid reduced the expression of all genes (*p* < 0.05), except for *bssS*, that was not different from the control, and *faeG*, which showed an increase in transcription levels (*p* < 0.05). Sorbic acid upregulated the expression of all the examined genes (*p* < 0.05), except for *faeG* and *bssS*. Finally, hexanoic acid was not different from the control, except for an increase of *bssS* mRNA levels (*p* < 0.05) (Figure 2C).

Considering the strong modulatory effect by NIC, gene expression results were pooled by treatment (NIC vs. AB) and compared. Results showed that the efficacy of NIC was the same as that of AB (*p* > 0.05).

## 3. Discussion

One of the most striking issues in the worldwide pig industry is represented by PWD. Despite recent advances, ranging from improved breeding conditions to new strategies to control infectious diseases, PWD still constitutes a significant challenge. Antibiotics represent a major strategy to manage infections by *E. coli* K88, identified as the main etiological agent for PWD [1,18].

In this study, an *E. coli* K88 field strain was tested against a panel of several antibiotics widely used to manage livestock infections. The strain was resistant to the vast majority of them, with colistin and doxycycline not only being the only two effective molecules in inhibiting the growth of *E. coli*, but also in repressing virulence gene expression, especially for genes encoding bacterial LT and ST toxins, primarily responsible for the onset of diarrheic symptoms in pigs.

In an extensive analysis by Klein et al., more than 200 *E. coli* strains across eight European countries were assessed for their resistance against dozens of antibiotics: results showed a considerable resistance towards antimicrobial compounds, including amoxicillin [19]. Other studies reported similar results, with MIC values for different *E. coli* strains equal to or greater than 64 mg/L for neomycin, ampicillin, and tetracyclines, proving an established or increasing spread of resistance genes [20,21,22,23]. *E. coli* resistance to penicillin G and lincomycin is also reported [20]. Colistin is one of the few antibiotics employed both in human and veterinary medicine and it is considered as a last resort for infections by multi-drug resistant Gram-negative species [24]. Its use in the pig industry allows the control of *E. coli* infections with a certain degree of effectiveness [25]. However, the appearance of the *mcr-1* colistin resistance gene is a serious threat to the efficacy of this primary importance antibiotic [26], therefore European authorities encouraged member states to support initiatives oriented towards a significant reduction in its use [27]. Our study showed that colistin was effective at an MIC of 4 mg/L, a value considered to be a sign of antimicrobial resistance, according to EUCAST breakpoints [28].

The spread of multi-resistant bacteria caused by antimicrobials use and abuse, together with the difficulties in finding new antibacterial drugs [29], dictates that novel strategies to manage *E. coli* K88 infections are required. OA and NIC have gained attention because of their recognized antimicrobial properties [13,14] and could therefore represent a valid approach to help manage bacterial infections as stand-alone treatments or as antibiotic adjuvants [9,30]. 

In this study, a large number of NIC and OA was tested against *E. coli* K88, proving the efficacy of three phenol derivatives of terpenes—i.e., eugenol, thymol, and its isomer carvacrol—and the growth-inhibiting activity of sorbic acid, benzoic acid, and hexanoic acid. Thymol, carvacrol, and eugenol were also tested (individually or as essential oils) in many previous studies which reported the positive effects of these bioactives against several *E. coli* strains both in vivo and in vitro [15,31,32,33]. Moreover, several in vivo studies demonstrated the usefulness of OA in controlling PWD in orally challenged piglets [34,35,36,37].

The target of *E. coli* K88 is represented by intestinal mucosa cells: the bacterium needs close contact to the epithelial cells to exert its pathogenicity [38,39]. For this reason, the flagellar movement is vital for *E. coli* colonization of the hindgut and can also help the F4 fimbrial adhesin to bind cellular targets [40]. Our study revealed that selected antibiotics, NIC, and benzoic acid downregulate the expression of *motA* and *faeG* genes crucial for bacterial motility and cellular adhesion. The same trend was registered on *E. coli* O157:H7: an important downregulation of genes related to flagella was shown for bacteria conditioned with sub-MIC doses of thymol and carvacrol [41]. 

Moreover, in this study, effective antibiotics, selected NIC, and benzoic acid demonstrated a significant ability to downregulate the expression of genes encoding for LT, STa, and STb enterotoxins, the true heart of ETEC pathogenicity. To the best of our knowledge, this was the first study that not only proved the efficacy of thymol, carvacrol, eugenol, and benzoic acid against bacterial proliferation, but also demonstrated the capacity of these compounds to downregulate the expression of the three *E. coli* K88 major enterotoxin genes.

The same molecules also affected *E. coli* genetic machinery involved in quorum sensing (QS) and biofilm formation via downregulation of *luxS*, a gene encoding for a lyase that synthesizes a precursor of the QS autoinducer-2 [42], but also *bssS* and *tnaA*, two genes involved in the regulation of biofilm formation [43,44,45]. Taken all together, these data suggest a diversified mode of action of these molecules that seems to be exploited by a complex series of reactions, rearrangements, and influences of the genetic apparatus of *E. coli*. 

Globally, while NIC seem to share a common pathway in downregulating *E. coli* K88 virulence genes, sub-lethal concentrations of OA evidenced three differential behaviours on transcription modulation, despite their comparable ability to inhibit bacterial growth at higher doses. While thymol, carvacrol, and eugenol all have a common chemical structure—as they all share traits of aromaticity and hydrophobicity—organic acids, except for benzoic, have different chemical properties and represent a more diverse group of compounds. Thymol, carvacrol, eugenol, and benzoic acid all share an aromatic benzene ring with polar bioactive groups. These similarities in structure, as well as their high hydrophobicity, would suggest that their mode of action might be connected to their capability of diffusing through the cell membrane and causing impairment to normal membrane functions [46,47]. Moreover, their aromatic rings, naturally able to delocalize electrons, can contribute to the reduction of membrane proton gradient, thus resulting in a collapse of the proton motive force and the depletion of ATP [47,48], with a potential consequence even on virulence gene expression levels because of an extensive effect on several bacterial enzymes [15], finally resulting in an impairment of virulence functions such as the gene expression of bacterial toxins like LT, STa, and STb. This would also suggest a mechanism of action of these compounds similar to certain antibiotics like colistin, doxycycline, and bacitracin, for example, that would implicate a possible synergy between these categories of molecules as a new frontier of therapeutic approach against enterotoxin-producing pathogens [9,49,50,51,52,53,54].

On the other side, hexanoic and sorbic acid were not equally effective as benzoic in downregulating *E. coli* gene expression despite their inhibitory activity in MIC and MBC tests. Hexanoic acid was ineffective concerning modulating the virulence genes, as its mechanism of action is mostly directed against Gram-positive bacteria [55,56], whereas sub-MIC concentrations of sorbic acid, which is equally defined as a medium chain fatty acid with six atoms of carbons, increased virulence gene expression despite its relatively high bactericidal activity: mild acid-stress conditions generated by sorbic acid in *Bacillus subtilis* can activate the ppGpp-dependent stringent response and an acid shock response mediated by RelA and Fur regulons [57,58], conditions that also strengthen virulence gene expression in *E. coli* [59,60].

This evidence further suggests that the antimicrobial action and the ability to modulate *E. coli* K88 virulence genes are two independent properties of OA, and are likely not related to each other. This observation remarks on the importance of the inclusion of a correct OA dose in animal diets to control bacterial infections, also with the aid of protecting techniques such as microencapsulation [13], to avoid a dangerous undesired upregulation of several virulence genes like toxin-related ones.

## 4. Conclusions

Our data show that bioactive molecules like nature-identical compounds (NIC) and organic acids (OA) can exert a strong antimicrobial power against *Escherichia coli* K88 (*E. coli* K88). While sub-inhibitory concentrations of OA have a diverse effect on virulence genes modulation, sub-lethal concentrations of select NIC have a strong impact on the expression of *E. coli* K88 virulence genes, especially on heat-labile (LT) and heat-stable (ST) toxins, mainly responsible for diarrhoea onset in weanling piglets. Furthermore, in this study, the extent of the efficacy and the mode of action of such molecules was comparable to that of colistin and doxycycline.

In conclusion, NIC and OA can represent a valid tool to partially replace and/or complement antibiotic treatment in post-weaning diarrhoea management and enterotoxigenic *E. coli* K88 infection control.

## 5. Materials and Methods

### 5.1. Bacterial Strain and Culture Conditions

The bacterium used in this study was *Escherichia coli* K88 (*E. coli* K88), a field strain originally obtained from the intestine of a piglet with post-weaning diarrhoea. The frozen culture was activated and routinely cultured in a brain heart infusion broth (BHI; VWR International Srl, Milan, Italy) (pH 6.5) at 37 °C in aerobic conditions and counted via plating 10-fold serial dilutions onto BHI agar. Daily 1:100 passage was performed to maintain an active *E. coli* K88 culture.

### 5.2. Chemicals and Stock Solutions

The antibiotics (AB) used in this study were amoxicillin, ampicillin, doxycycline, lincomycin, neomycin, penicillin G, and colistin (all obtained from Alpha Aesar, Thermo Fisher GmbH, Kandel, Germany); AB stock solutions were prepared in BHI. Organic acids (OA) and nature-identical compounds (NIC) utilized in this study were citric acid, sorbic acid, benzoic acid, butyric acid, hexanoic acid, formic acid, fumaric acid, lactic acid, malic acid, and propionic acid (stocks prepared in BHI); and octanoic acid, decanoic acid, dodecanoic acid, thymol, carvacrol, eugenol, vanillin, α-pinene, eucalyptol, limonene, linalool, and menthol (stocks prepared in BHI supplemented with ethanol at a final concentration ≤ 3.5% to increase solubility); all OA and NIC were obtained from Merck KGaA, Darmstadt, Germany. Each solution was buffered to ensure a final pH of 6.5, filter-sterilized and diluted in sterile BHI to reach the final concentration tested.

### 5.3. Minimal Inhibitory Concentration (MIC) Assay

MIC of AB, OA, and NIC was determined using the microdilution method in 96-well microtiter plates [61]. *E. coli* K88 was tested against a wide range of concentrations of all the selected compounds: antibiotics (0.5–64 mg/L), citric acid, sorbic acid, benzoic acid, butyric acid, hexanoic acid, formic acid, fumaric acid, lactic acid, malic acid, and propionic acid (1.56–100 mM); octanoic acid, decanoic acid, dodecanoic acid, thymol, carvacrol, eugenol, vanillin, α-pinene, eucalyptol, limonene, linalool, and menthol (0.12–7.5 mM). The bacterial strain (10^5^ CFU/mL) was incubated with the tested substances at 37 °C for 24 h in aerobic conditions. Control strain was grown in BHI supplemented with 3.5% ethanol to exclude inhibitory effects exerted by the ethanol eventually contained in stock solutions. After incubation, the 630 nm absorbance was read at the spectrophotometer (Varioskan™ LUX Multimode Microplate Reader, Thermo Fisher Scientific Inc., Waltham, MA, USA) to measure bacterial growth. The MIC value was defined as the lowest concentration of each compound capable to zero the absorbance (i.e., the bacterial growth) after 24 h of incubation.

### 5.4. Minimal Bactericidal Concentration (MBC) Assay

Minimal bactericidal concentration (MBC) assays were performed for those compounds that reported a MIC value in the MIC assay (i.e., doxycycline, colistin, thymol, carvacrol, eugenol, sorbic acid, benzoic acid, and hexanoic acid). The MBC of AB, OA, and NIC was determined using the microdilution method in 96-well microtiter plates [62]. The bacterial strain (10^5^ CFU/mL) was incubated for 24 h at 37 °C in aerobic conditions with the following compounds: doxycycline and colistin (1–64 mg/L), thymol, carvacrol, and eugenol (0.12–15 mM), sorbic acid, benzoic acid, and hexanoic acid (1.56–200 mM), respectively. Control strain was grown in BHI supplemented with an adequate amount of ethanol to exclude bactericidal effects exerted by the ethanol eventually contained in stock solutions. After incubation, the 630 nm absorbance was read at the spectrophotometer (Varioskan™ LUX Multimode Microplate Reader, Thermo Fisher Scientific Inc., Waltham, MA, USA) to confirm the MIC, while samples taken from limpid wells were plated on BHI agar plates and incubated overnight at 37 °C in aerobic conditions to assess MBC. The MBC value was defined as the lowest concentration of each tested compound capable to avoid the recovery of bacterial colonies after seeding and enumeration of limpid wells on BHI agar plates.

### 5.5. Gene Expression Analysis

Gene expression analysis was performed on bacterial cells adapted in BHI containing antimicrobial compounds. For the substances that reported an MBC value, sub-MBC (half of MBC) concentrations were used. Instead, for hexanoic acid and benzoic acid that did not report an MBC, a sub-MIC (half of MIC) concentration was chosen. For amoxicillin, the highest tested concentration was chosen (64 mg/L). To prepare adapted bacteria, 10^6^ CFU/mL of an overnight *E. coli* K88 culture were grown at 37 °C for 4 h after inoculation into 5 mL of fresh BHI supplemented with either 32 mg/L doxycycline, 2 mg/L colistin, 64 mg/L amoxicillin, 0.94 mM thymol, or carvacrol, 1.87 mM eugenol, 25 mM sorbic acid, benzoic acid, or hexanoic acid. Adequate controls were prepared for substances containing ethanol in their stock solutions. 

After incubation, bacterial cells were collected by centrifuging 5 min at 5000× *g*. After supernatant removal, the pellet was resuspended in 100 μL of Tris-EDTA buffer supplemented with 1 mg/mL of lysozyme and incubated at 37 °C for 10 min. Then, total RNA extraction from bacteria was performed using the NucleoSpin RNA Kit (Macherey-Nagel GmbH & Co. KG, Düren, Germany) with DNase digestion according to manufacturer’s instructions. 

RNA yield and quality were verified spectrophotometrically using A230, A260, and A280 nm measurements (μDrop Plate and Varioskan LUX, Thermo Fisher Scientific Inc., Waltham, MA, USA).

Four hundred ng of RNA were subsequently reverse-transcribed with iScript cDNA Synthesis Kit (Bio-Rad Laboratories, Inc., Hercules, CA, USA) according to manufacturer’s instruction. The cDNA obtained was used as the template for qPCR analysis, whose reactions were prepared in a final volume of 10 μL, containing 5 μL of 2x iTaq Universal SYBR Green Supermix (Bio-Rad Laboratories, Inc., Hercules, CA, USA), 200 (for *faeG*, *eltB*, *bssS*, *tnaA*, and *16S*) or 600 nM (for *eltA*, *estA*, *estB*, *motA*, *luxS*, and *ihfB*) of each primer, 2 μL of 5 ng/μL cDNA, and nuclease-free water up to volume. Real-time PCR was performed using CFX96 Real-Time PCR Detection System (Bio-Rad Laboratories, Inc., Hercules, CA, USA) under the following conditions: 3 min at 95 °C, followed by 40 cycles of 95 °C for 10 s and 60 °C for 30 s. The specificity of each reaction was evaluated by melting-curve analysis with 0.5 °C/s heating rate from 55 up to 95 °C. 

Primer specificity was evaluated by Sanger sequencing of control qPCR products (Microsynth AG, Balgach, Switzerland) after purification with NucleoSpin Gel and PCR Clean-up Kit (Macherey-Nagel GmbH & Co. KG, Düren, Germany). All sequencing products were considered valid only if matching the expected amplicon.

Gene expression was normalized using two reference genes, i.e., the 16S rRNA gene (*16S*) and the B subunit of the integration host factor (*ihfB*). After determining the threshold cycle (Ct) for each gene, the relative changes in gene expression of *E. coli* K88 grown in media supplemented with treatments compared to controls were calculated using the 2^−ΔΔCt^ method [63].

Forward (F) and reverse (R) primers (Table 2) were designed using Primer-BLAST tool (NCBI; National Center for Biotechnology Information) and synthesized by Merck KGaA, Darmstadt, Germany. 

### 5.6. Statistical Analysis

For MIC and MBC assays, experiments were performed in three technical replicates and data are presented as means. For gene expression analysis, experiments were performed in triplicate (n = 3) and data are presented as means ± SEM. Gene expression data were analyzed with GraphPad Prism v. 8.4.1 (GraphPad Software, Inc., San Diego, CA, USA) performing One-Way ANOVA with Tukey post hoc test. Comparisons between the overall effect of groups of substances were performed by pooling the results of gene expression analysis by treatment and performing the Mann–Whitney test amongst groups. Differences were considered significant at *p* ≤ 0.05.

## Figures and Tables

**Table 1:** Minimal inhibitory concentration (MIC) of antibiotics (AB), nature-identical compounds (NIC), and organic acids (OA) against *E. coli* K88.

Antibiotics (AB)		Nature-Identical Compounds (NIC)		Organic Acids (OA)	
Substance	MIC (mg/L)	Substance	MIC (mM)	Substance	MIC (mM)
Amoxicillin	>64	α-pinene	>7.5	Benzoic	50
Ampicillin	>64	Carvacrol	1.87	Butyric	>100
Colistin	4	Eucalyptol	>7.5	Citric	>100
Doxycycline	32	Eugenol	3.75	Decanoic	>100
Lincomycin	>64	Limonene	>7.5	Dodecanoic	>100
Neomycin	>64	Linalool	>7.5	Formic	>100
Penicillin G	>64	Menthol	>7.5	Fumaric	>100
		Thymol	1.87	Hexanoic	25
		Vanillin	>7.5	Lactic	>100
				Malic	>100
				Octanoic	>100
				Propionic	>100
				Sorbic	50

**Table 2:** Primers used in this study for real-time PCR.

Function	Gene	Sequence (5′ → 3′)	Product Length (bp)	AN ^1^	Ref ^2^
Adhesion to cells	*faeG*	F: ACGTCGCAGGTTCTTACAGG R: GCTCCACTGAGTGCTGGTAG	140	M35954	This study
LT toxin production	*eltA*	F: TTGGTGATCCGGTGGGAAAC R: AGGAGGTTTCTGCGTTAGGTG	185	LN870273	This study
	*eltB*	F: CACGGAGCTCCCCAGACTAT R: GCCTGCCATCGATTCCGTAT	105	M17873	This study
STa toxin production	*estA*	F: CAACTGAATCACTTGACTCTT R: TTAATAACATCCAGCACAGG	158	V00612	[64]
STb toxin production	*estB*	F: TGCCTATGCATCTACACAA R: CTCCAGCAGTACCATCTC	113	M35586	[64]
Flagellar movement	*motA*	F: TGAACGACCCCCATTACAGC R: AGCGGTCACATGAACACCTT	155	NZ_LBBN01000002	This study
Quorum sensing	*luxS*	F: CAGTGCCAGTTCTTCGTTGC R: TGAACGTCTACCAGTGTGGC	116	HQ538844	This study
Biofilm formation	*bssS*	F: TCCCTTCCTGCTCGGACTTA R: CAGACTCATCCGCTCGTAGG	106	NZ_LBBN01000031	This study
	*tnaA*	F: CGCCAAGAAAGATGCGATGG R: CGTCATACAGACCTACCGCC	173	NZ_LBBN01000006	This study
Housekeeping genes	*ihfB*	F: CCCGTCAAGACGGTTGAAGA R: TCGCCAGTCTTCGGATTACG	152	NC_017641	This study
	*16S*	F: GAGGGCGCTTACCACTTTGT R: GTAAGGAGGTGATCCAACCG	90	NC_017641	This study

^1^ AN = Accession Number; ^2^ Ref = Reference.
